# Evaluation of the effect of kidney transplantation on left ventricular myocardial work by noninvasive pressure-strain loops

**DOI:** 10.3389/fcvm.2024.1370307

**Published:** 2024-07-25

**Authors:** Zhengyang Han, Lingyun Wang, Honghu Wang, Hongying He, Yi Song, Menghe Wang, Na Zhao, Zhengguang Chen, Zhenxing Sun, Shan Zhang

**Affiliations:** ^1^ Department of Ultrasound, The First Affiliated Hospital of Zhengzhou University, Zhengzhou University, Zhengzhou, Henan, China; ^2^ Department of Ultrasound Medicine, Union Hospital, Tongji Medical College, Huazhong University of Science and Technology, Wuhan, China

**Keywords:** kidney transplantation, echocardiography, myocardial work, three-dimensional speckle tracking echocardiography, pressure-strain loops

## Abstract

**Purpose:**

Kidney transplantation (KT) has the potential to reverse the cardiac changes caused by end-stage renal disease, and it may be inaccurate to analysis the left ventricular function by conventional echocardiography due to afterload. This study aimed to investigate the utility of pressure strain loops (PSLs) in evaluating left ventricular performance in patients underwent KT.

**Methods:**

We enrolled 60 patients with end-stage renal disease who underwent KT between January 2022 and July 2023, and 60 healthy controls with a similar distribution of gender and age to the patients. All participants underwent conventional echocardiography and three-dimensional speckle tracking echocardiography (3D-STE). Long axis, short axis, and four cavity images were collected and cardiac parameters were measured. The echocardiographic changes of cardiac structure and function of all patients before KT and about 12 months after KT were recorded. Left ventricular myocardial work parameters were acquired by PSLs, including the global work index (GWI), global constructive work (GCW), global wasted work (GWW), global work efficiency (GWE) and global longitudinal strain (GLS). In addition, the correlation between PSLs and clinical data were explored.

**Results:**

Compared with controls, the conventional echocardiographic parameters, myocardial function indicators GWI and GCW appeared no difference in post-KT group, while the GWE and GLS decreased (*p *< 0.05), and the GWW increased (*p *< 0.05). Compared with pre-KT, the GLS, GWI, GCW and GWE increased in post-KT group, while the GWW decreased (all *p *< 0.05). The above indicators were correlated with left ventricular GLS and left ventricular ejection fraction.

**Conclusion:**

PSLs were more sensitive than traditional echocardiographic indicators in detecting changes in myocardial work and predicting left ventricular myocardial damage. This indicator could quantitatively evaluate myocardial work and provide a new and reliable non-invasive reference for clinical diagnosis and treatment of patients underwent KT.

## Introduction

Kidney transplantation (KT) is an effective treatment for end-stage renal disease (ERSD), which can reduce the main consequences of renal failure and promote restoration of organ function (1–3). KT has been reported that it could reduce cardiac mortality and the risk for development of chronic heart failure compared with long-term dialysis (4). However, some studies have shown that cardiovascular diseases account for 31% of patient deaths with a functioning graft (5). Although transthoracic echocardiography is regarded as the most reliable noninvasive predictor of elevated LV function in patients with ESRD, there is limited ability in diagnosising subclinical myocardial injury without morphological and functional changes (6). The myocardial strain index based on three-dimensional speckle tracking echocardiography (3D-STE) could provide a sensitive detection method for subclinical myocardial function, but it has load dependency (7). Pressure strain loops (PSLs) originate from 3D-STE, which not only maintains the sensitivity and repeatability of 3D-STE, but also overcomes the dependence on afterload (8). Research has confirmed a strong correlation between PSLs and local myocardial glucose metabolism, which can objectively evaluate myocardial oxygen consumption and systolic function (9). In recent years, PSLs have been widely used in evaluating left ventricular myocardial function and the efficacy of cardiac resynchronization therapy in cardiomyopathy both domestically and internationally, and their reliability has been verified in various cardiovascular diseases, allowed an earlier and more sensitive detection of subtle changes of myocardial function (10, 11). However, PSLs have rarely been applied in the evaluation of cardiac function after KT (12). Hence, the aim of this study was to assess the changes in myocardial work on PSLs before and 1 year after successful KT. Furthermore, we sought to describe the possible impact of KT on left ventricular myocardial mechanics.

## Methods

The study was conducted at the First Affiliated Hospital of Zhengzhou University, Zhengzhou, China. It was approved by the ethics committee of the university, and was carried out in accordance with the Declaration of Helsinki after written informed consent was obtained from all patients (13). All KT operations were performed with the permission of the National Authorization Committee, and the kidneys were from living donors.

### Study population

60 patients with end-stage renal disease on chronic hemodialysis were enrolled, who subsequently underwent KT surgery in the aforementioned study site between January 2022 and July 2023. The inclusion criteria contained: (a) ages between 18 and 60; (b) no segmental wall motion abnormality and/or end-stage heart failure; (c) the images were clear and easy to analyze by 3D-STE; and (d) heart rate between 60 and 100 bpm, sinus rhythm. All selected patients had informed consent to this experimental study. Exclusion criteria were as follows: (a) suspicion of acute graft rejection; (b) combined with coronary artery disease (a stenosis >50% in any major artery or distal pruning of any secondary branch based on coronary computer tomography angiography) and/or valvular heart disease (moderate-to-severe valvular regurgitation or stenosis); (c) receiving double organ transplantation (heart-kidney transplantation, liver-kidney transplantation and so on); (d) within 12 months post-KT; and (e) hypertensive patients taking GDMT-type anti-hypertensive medications (14).

The control group consisted of 60 healthy volunteers in the same period, with a similar distribution of sex and age to the KT patients. They had no cardiac chest pain, vasodepressor syncope, functional heart murmurs or anatomic heart disease.

### Clinical data collection

The medical history, duration of end-stage renal disease, complications, and treatment status of the participants were recorded in detail. Laboratory testing was conducted on all participants, and echocardiography was performed before and after KT, while blood pressure was measured at the same time. Other measures included age, gender, height, weight, body mass index (BMI), heart rate, smoking, hypertension and diabetes. Patients after KT generally received formal immunosuppressive regimens, including calcineurin inhibitor (CNI), induction therapy (Anti-interleukin-2 receptor antibody), steroids and mycophenolate. The included patients have overcome acute allograft rejection confirmed by biopsy. If there was no rejection after half a year, steroids could be gradually reduced (4). Blood samples were collected from cubital veins before and after KT. And standard laboratory parameters were obtained from blood and serum containing EDTA after centrifugation. Spontaneous urinary protein excretion was quantified by turbidimetric protein assay with the biuret method, and glomerular filtration rate (GFR) was calculated refering from the modification of diet in renal disease equation (15). The medication of patients in the experimental group before and after KT was recorded, including immunosuppression, glucocorticoids and antihypertensive drugs.

### Conventional echocardiography

The standard transthoracic echocardiography examination was performed in all participants using the GE Vivid E95 ultrasound machine (GE medical systems) with an M3S transducer (1–5 MHz). Echocardiographic parameters were obtained in accordance with the current recommendations of the American Society of Echocardiography (16). All images were acquired without anesthesia and sedation during a single breath-hold. The images were collected from the apical four chamber heart, three chamber heart and two chamber heart sections, and the 2D dynamic images of the subject were continuously collected for 4 cardiac cycles. Conventional chamber sizes and wall thickness were measured according to the method recommended by American Echocardiography Association (17). The left atrial diameter end systolic (LADs), left ventricular internal diameter diastolic (LVIDd), left ventricular internal diameter systolic (LVIDs), interventricular septal thickness (IVST) and left ventricular posterior wall thickness (LVPWT) were measured in the long axis section of the LV. Peak early diastolic flow velocity (E), end diastolic flow velocity (A) of LV, their ratio E/A, deceleration time (DT) and early diastolic tissue velocity at the mitral annular septum (e') were measured in the apical four chamber views, and calculate E/e'. Simpson method was used to calculate left ventricular end-diastolic volume (EDV), end-systolic volume (ESV), left ventricular fractional shortening (LVFS) and left ventricular ejection fraction (LVEF).

### Myocardial work

For the LV function analysis, the original data images were copied to the EchoPAC workstation for analysis. This method could associate 3D volume acquisition with time-curve derived 3D deformation analysis, so that allow the automatic tracking of the whole volume of a given cardiac cavity over time and the generation of a time-volume curve as well as phasic deformation data. The software could automatically outline the endocardial boundary, which was manually adjusted in position and width to coincide with the left ventricular myocardial range (18). After that, the system will automatically provide the left ventricular global longitudinal strain (GLS). After entering the blood pressure value, the overall myocardial work parameters could be obtained, including global work index (GWI), global constructive work (GCW), the global wasted work (GWW) and global work efficiency (GWE). The calculation of myocardial strength was difficult, so left ventricular systolic pressure measurements were estimated based on brachial cuff blood pressure. Myocardial work analysis was measured during this systolic stroke work, or mechanical systole plus isovolumetric relaxation pressure, which began at mitral valve closure and ended at mitral valve opening and was expressed in units of mmHg%.

**Figure 1:**
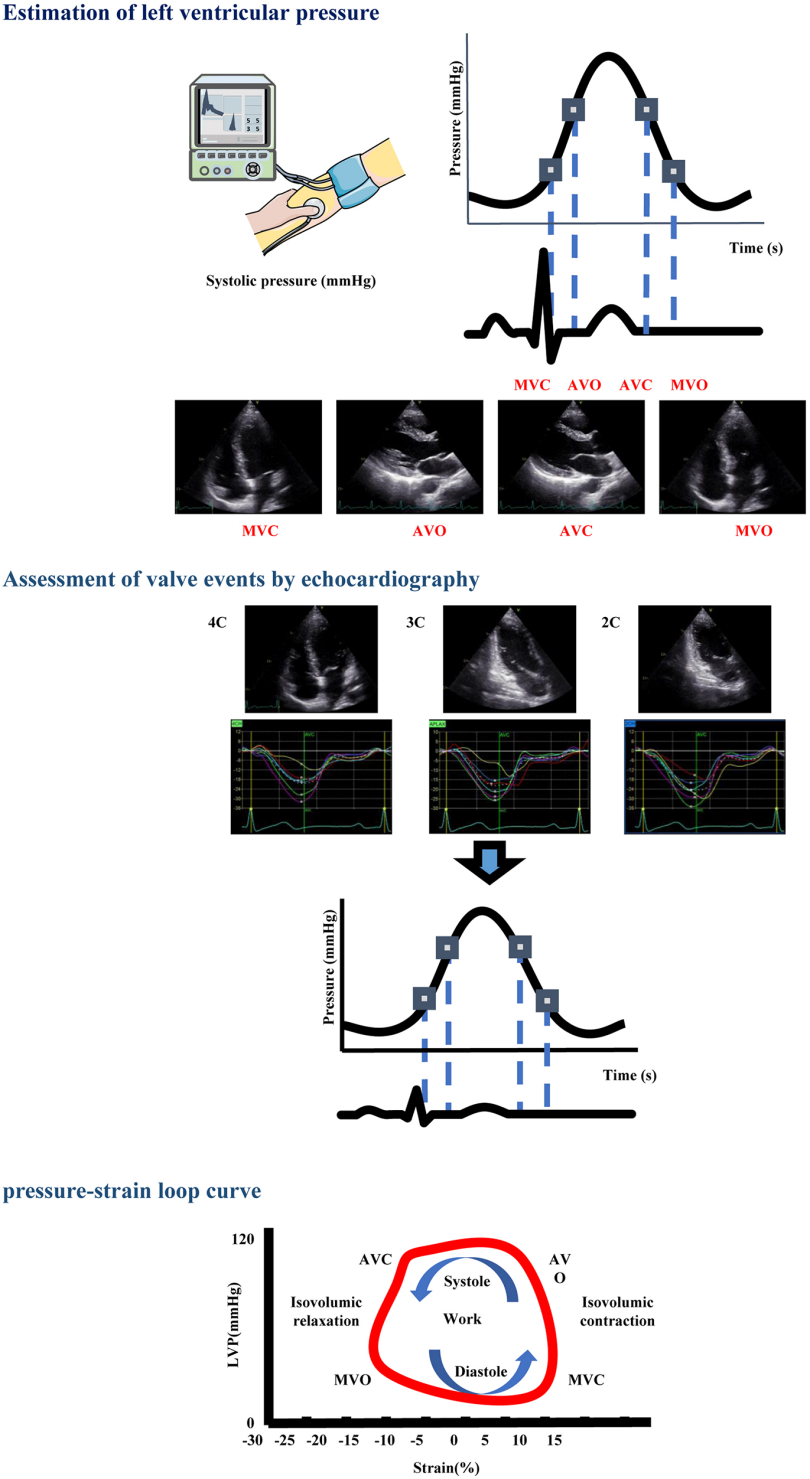
Operation method for obtaining PSL parameters. STEP 1: The referral LV pressure curve was scaled according to systolic brachial artery cuff pressure and valvular events assessed by echocardiography. STEP 2: Pressure curve and strain data were merged by the QRS peak as time-reference in order to obtain the LV PSL. The area within the PSL represents work performed by the left ventricle. The loop operates counterclockwise, showing the correlation between cardiac event times and the relationship between GLS and estimated left ventricular systolic blood pressure (brachial cuff blood pressure). AVC, aortic valve closure; AVO, aortic valve opening; MVC, mitral valve closure; MVO, mitral valve opening; LVP, left ventricular pressure.

Myocardial work measurement indicators included the followings:

(a) GWI: the total work of the left ventricle during the period from mitral valve closure to mitral valve opening plus isovolumic contraction and isovolumetric relaxation, which was reflected by the area within the PSLs.
(b) GCW: productive work performed by the left ventricle during systole, including both the muscle shortening during systole and the muscle lengthening in isovolumetric relaxation.
(c) GWW: unproductive work performed by the left ventricle during systole, including both the muscle lengthening during systole and the muscle shortening in isovolumetric relaxation.
(d) GWE: the ratio of constructive work to the sum of constructive and wasted work [GCW/(GCW + GWW)] (Figure 1) (19, 20).

### Statistical analysis

Statistical analysis was performed using IBM SPSS Statistics version 22.0 (SPSS, Inc). Continuous variables were presented as mean ± standard deviation (¯x ± s). Categorical variables were expressed as frequencies or percentages. Comparison of results with normal distribution was performed using student *t* test and analysis of variance as appropriate, pairwise comparison between groups was conducted using LSD-*t* test. Using nonparametric testing for data with non normal distribution. The comparisons between the control group, pre-KT group and post-KT group were performed using 1-way analysis of variance, and ANOVA with *post-hoc* comparisons was adopted for pairwise comparison between each group. Spearman correlation (non normal distribution) or pearson correlation (normal distribution) tests were conducted to test the association between parameters. Intra- and inter-observer variability for myocardial work parameters were assessed by intraclass correlation coefficients (ICCs) analysis. A 2-sided *p* value < 0.05 was considered to indicate statistical significance.

## Results

### Baseline parameters

The study enrolled 60 patients with end-stage renal disease who underwent KT. The etiology of end-stage renal disease included chronic glomerulonephritis (46.7%), diabetic nephropathy (18.3%), hypertensive nephropathy (25.0%), chronic tubulointerstitial disease (5.0%) and polycystic kidney disease (5.0%). 10 cases smoked, 6 cases had diabetes and 18 cases suffered from hypertension (according to 2020 International Society of Hypertension Global Hypertension Practice Guidelines) (21). The median elapsed time since KT was 12.8 (12.0–15.4) months. During postoperative follow-up, the mean GFR was (59.9 ± 12.9) ml/min, and the median spontaneous urinary protein excretion was (75.4 ± 35.7) mg/L. There was no significant difference in the dosage of antihypertensive drugs before and after KT, and no cardiovascular events occurred. The baseline characteristics of the KT and control groups were shown in Table 1, and the medication of patients before and after KT was recorded in Table 2.

**Table 1:** Baseline patient characteristics in KT group and control group [*n* (%) or (¯x ± s)].

Variable	KT group (*n* = 60)	Control group (*n* = 60)	*t*/*χ*^2^	*p*
Age (year)	42.5 ± 15.1	40.3 ± 14.5	0.809	0.420
Male [*n*(%)]	32 (53.3)	28 (46.7)	0.533	0.465
Height (cm)	167.4 ± 7.7	169.1 ± 8.5	1.158	0.249
Weight (kg)	65.5 ± 12.4	63.9 ± 10.5	0.745	0.457
BMI (kg/m^2^)	23.4 ± 3.9	22.4 ± 3.8	1.368	0.174
Heart rate (bpm)	75.5 ± 7.4	73.3 ± 7.5	1.620	0.108
Systolic BP (mm Hg)	133.8 ± 17.3	128.3 ± 14.0	1.921	0.057
Diastolic BP (mm Hg)	86.9 ± 12.1	89.3 ± 15.2	0.946	0.346
Smoke [*n*(%)]	16 (26.7)	14 (23.3)	0.178	0.673
Diabetes [*n*(%)]	7 (11.7)	4 (6.7)	0.901	0.343

BMI, body mass index; BP, blood pressure.

**Table 2:** General medication of participants before and after KT [*n* (%) or (¯x ± s)].

Variable	Pre-KT (*n* = 60)	Post-KT (*n* = 60)	*t*	*p*
Average no. of pills of antihypertensive	3.0 ± 1.4	2.6 ± 1.2	1.707	0.090
ASA, *n* [%]	9 (15.0)	9 (15.0)		
ACE inhibitors, *n* [%]	15 (25.0)	17 (25.0)		
Angiotensin-receptor blockers, *n* [%]	8 (13.3)	18 (30.0)		
Alpha-receptor blockers, *n* [%]	5 (8.3)	7 (11.7)		
Beta-blockers, *n* [%]	18 (30.0)	17 (28.3)		
Calcium-channel blockers, *n* [%]	10 (16.7)	10 (16.7)		
Diuretics, *n* [%]	11 (18.3)	12 (20.0)		
Mycophenolate, *n* [%]	8 (13.3)	1 (1.7)		
Anti-interleukin-2 receptor antibody, *n* [%]	/	60 (100)		
Calcineurin inhibitor, *n* [%]	/	60 (100)		
Steroids, *n* [%]	/	60 (100)		
Belatacept, *n* [%]	/	3 (5.0)		

ACE, angiotensinconverting enzyme; ASA, acetylsalicylic acid.

### Conventional echocardiographic parameters

2D echocardiographic and Doppler findings of the left heart were presented in Table 2. Compared with the pre-KT group, the LVFS, LVEF and E/A increased in the post-KT group, while the LADs, LVIDd, LVIDs, E/e, EDV, ESV and DT decreased (all *p *< 0.05). Compared with the control group, there was no significant difference in all the above indicators in the post-KT group (all *p *> 0.05). The above changes reflected the improvement of cardiac systolic and diastolic function after KT. And the interventricular septum thickness and posterior wall thickness showed no differences in the three groups (all *p *> 0.05) (Table 3).

**Table 3:** Conventional echocardiographic parameters in pre-KT, post-KT and control group (¯x ± s).

Variable	Control (*n* = 60)	Pre-KT (*n* = 60)	Post-KT (*n* = 60)	F/Z	*p*
LADs (mm)	34.6 ± 2.9	36.6 ± 3.9	35.1 ± 3.5*	5.396	0.005
LVIDd (mm)	47.5 ± 4.4	49.8 ± 3.4	47.9 ± 4.0*	5.644	0.004
LVIDs (mm)	31.4 ± 3.5	34.4 ± 3.0	32.1 ± 3.4*	13.799	<0.001
IVST (mm)	10.2 ± 1.7	10.8 ± 1.7	10.3 ± 2.0	1.795	0.169
LVPWT (mm)	10.2 ± 2.0	10.7 ± 1.8	10.2 ± 2.0	1.552	0.215
E/A	1.1 ± 0.3	0.9 ± 0.3	1.1 ± 0.3*	3.258	0.041
E/e	10.9 ± 2.2	12.2 ± 2.9	10.8 ± 2.6*	5.118	0.007
DT (ms)	142.4 ± 27.9	164.4 ± 26.6	150.0 ± 29.1*	9.699	<0.001
EDV (ml)	106.1 ± 22.4	117.7 ± 18.6	108.1 ± 21.0*	5.399	0.005
ESV (ml)	39.9 ± 10.7	49.4 ± 10.3	41.8 ± 10.3*	13.744	<0.001
LVFS (%)	33.8 ± 5.2	30.8 ± 4.8	33.1 ± 5.0*	5.711	0.004
LVEF (%)	62.2 ± 6.8	57.8 ± 6.9	61.1 ± 7.1*	6.661	0.002

LADs, left atrial diameter end systolic; LVIDd, left ventricular internal diameter diastolic; LVIDs, left ventricular internal diameter systolic; IVST, interventricular septal thickness; LVPWT, left ventricular posterior wall thickness; E, early transmitral flow velocity; A, late transmitral flow velocity; e, mean peak early diastolic myocardial annular velocity; DT, E peak deceleration time; EDV, end-diastolic volume; ESV, end-systolic volume; LVFS, left ventricular fractional shortening; LVEF, left ventricular ejection fraction. *p*-value: one-way analysis of variance.

* *p *< 0.05 between post-KT and pre-KT group.

### Myocardial work parameters

**Figure 2:**
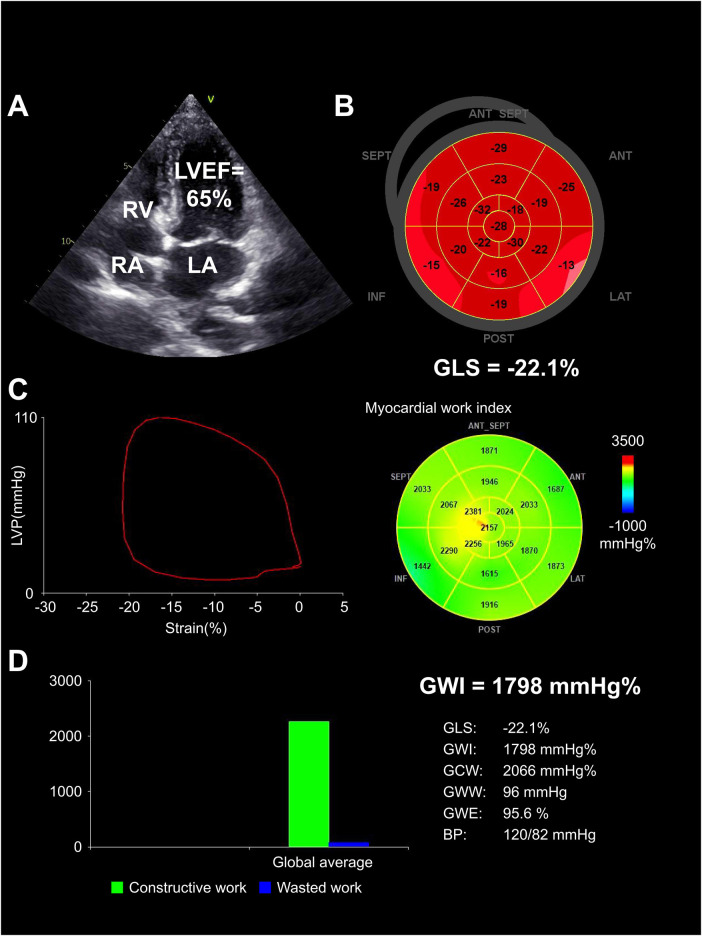
Structurally normal heart in control group. (**A**) Normal chamber size and LVEF (65%) demonstrated by echocardiogram. (**B**) Normal GLS (−22.1%), illustrated by completely red-shaded bull's-eye. (**C**) Normal GWI (1,798 mm Hg%), represented by the PSL and a predominantly green-colored bull's-eye. (**D**) Bar graph showed constructive work (green bar) vs. wasted work (blue bar) with corresponding numerical indices. LA, left atrium; RA, right atrium; RV, right ventricle.

**Figure 3:**
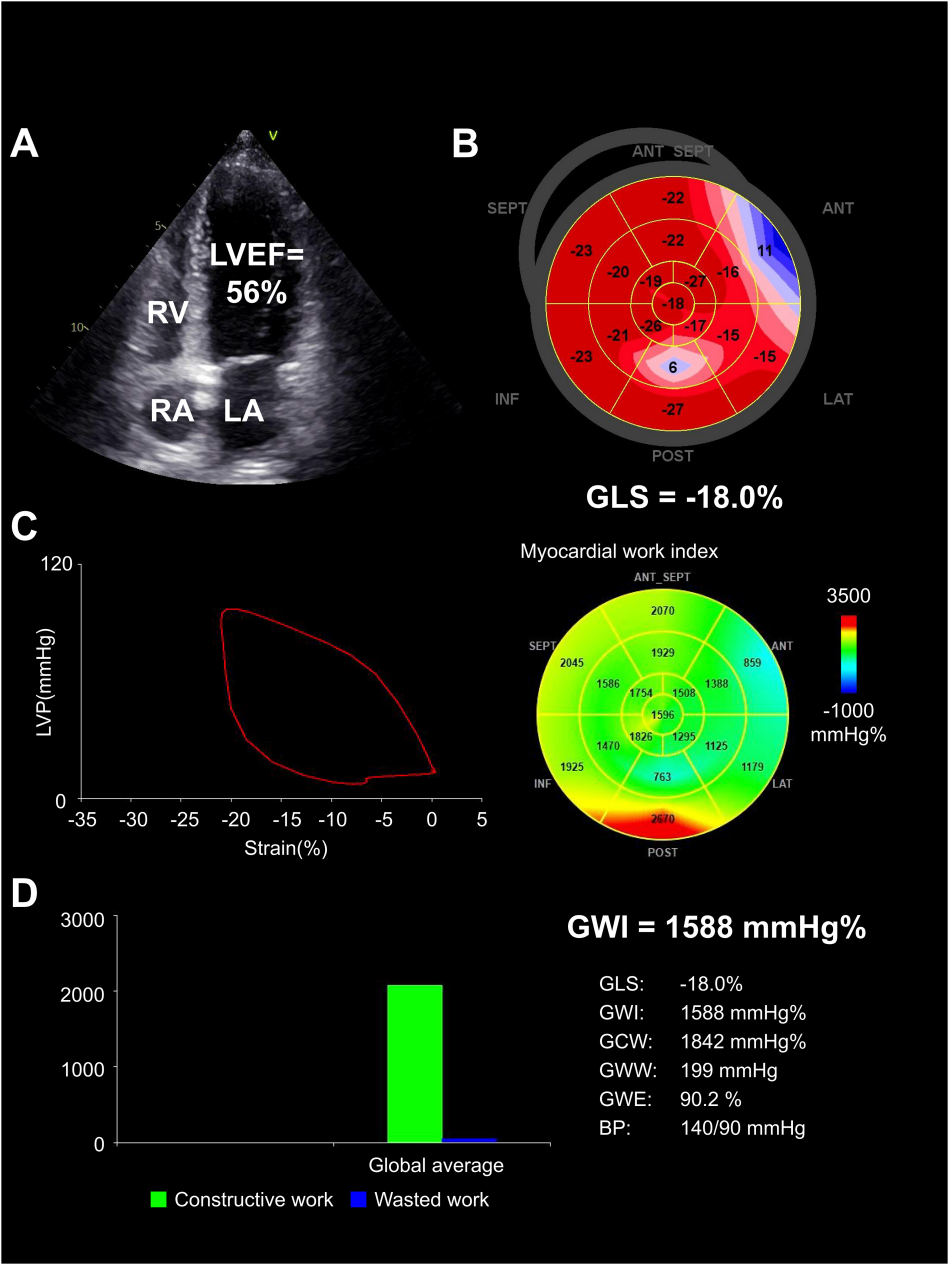
Heart in pre-KT group. (**A**) Echocardiographic findings demonstrated left chambers dilated, with decreased LVEF (56%). (**B**) GLS was severely reduced (−18.0%). (**C**) PSL and GWI was severely reduced (1,588 mm Hg%), displayed by a predominantly blue-colored bull's-eye. (**D**) Bar graph showed constructive and wasted work, with an increased amount of wasted work (199 mm Hg%) taking place. LA, left atrium; RA, right atrium; RV, right ventricle.

**Figure 4:**
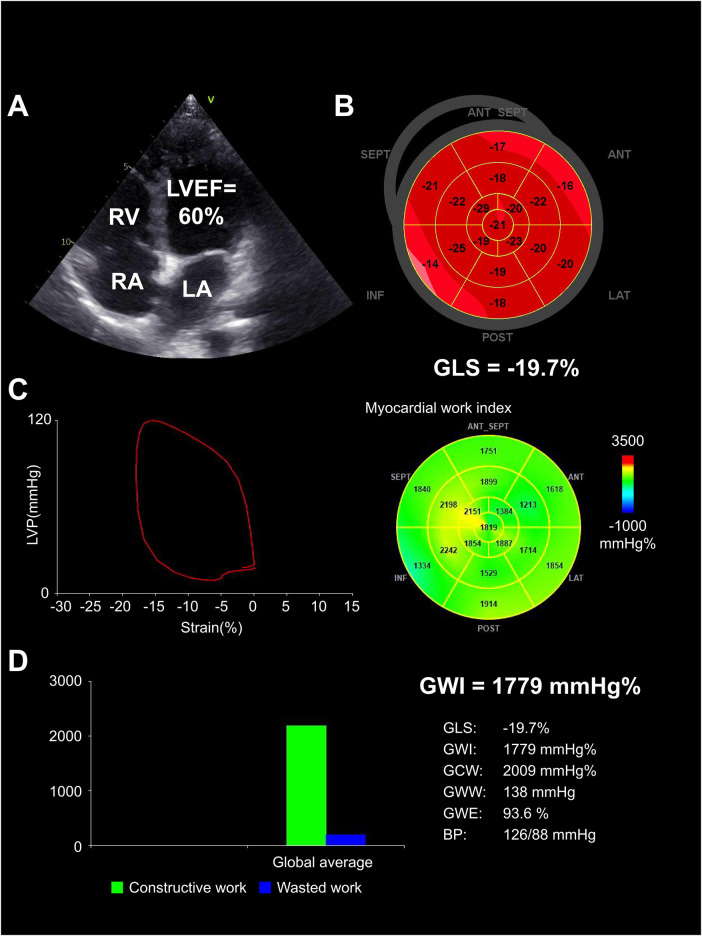
Heart in post-KT group. (**A**) Echocardiographic findings demonstrated the postoperative LVEF improved (60%), and the left ventricular cavity decreased compared to preoperative. (**B**) GLS increased compared to preoperative (−19.7%). (**C**) PSL showed an increase in GWI compared to preoperative (1,779 mm Hg%) (green segments). (**D**) Bar graph showed the recovery of relatively normal constructive and wasted work. LA, left atrium; RA, right atrium; RV, right ventricle.

Compared with pre-KT group, the post-KT group showed an increase in GLS, GWI, GCW, and GWE (*p *< 0.05), while the GWW decreased (*p *< 0.05). Compared with control group, the GWE and GLS decreased and GWW increased in post-KT group (all *p *< 0.05). There was no significant difference in GWI and GCW between the two groups (both *p *> 0.05) (Table 4, Figures 2–4).

**Table 4:** Myocardial work and GLS parameters in pre-KT, post-KT and control group (¯x ± s).

Parameters	Control (*n* = 60)	Pre-KT (*n* = 60)	Post-KT (*n* = 60)	F/Z	*p*
GWI (mmHg%)	1,778.7 ± 107.3	1,721.4 ± 130.9	1,764.2 ± 81.4*	4.538	0.012
GCW (mmHg%)	2,057.1 ± 305.7	1,860.3 ± 301.2	1,980.2 ± 361.0*	5.632	0.004
GWW (mmHg%)	165.1 ± 59.6	210.8 ± 61.6	186.9 ± 52.1*,**	9.329	<0.001
GWE (%)	92.5 ± 2.6	89.7 ± 3.1	91.2 ± 2.8*,**	14.379	<0.001
GLS (%)	−19.2 ± 3.6	−16.9 ± 2.6	−18.0 ± 3.2*,**	8.351	<0.001

GWI, global work index; GCW, global constructive work; GWE, global work efficiency; GWW, global wasted work; GLS, global longitudinal strain. *p*-value: one-way analysis of variance.

* *p *< 0.05 between post-KT and pre-KT group;

** *p *< 0.05 between post-KT and control group.

### Correlation analysis

**Figure 5:**
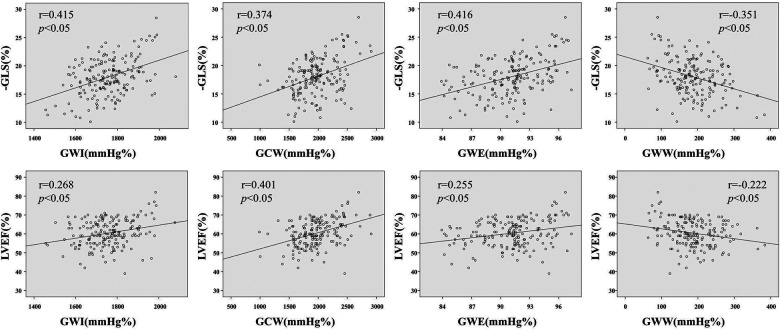
Correlation analysis between absolute values of GLS and LVEF and myocardial work parameters. GWI, global work index; GCW, global constructive work; GWE, global work efficiency; GWW, global wasted work; GLS, global longitudinal strain; LVEF, left ventricular ejection fraction; SP, systolic blood pressure; DP, diastolic blood pressure.

Correlation analysis demonstrated that GWI, GCW and GWE were positively correlated with the absolute values of GLS (*r* = 0.415, 0.374, 0.416, all *p *< 0.05) and LVEF (*r* = 0.268, 0.401, 0.255, all *p *< 0.05), while GWW was negatively correlated with the absolute values of GLS (*r* = −0.351, *p *< 0.05) and LVEF (*r* = −0.222, *p *< 0.05). There is no significant correlation between the above myocardial work indicators and blood pressure (all *p *> 0.05) (Table 5, Figure 5).

**Table 5:** Correlation analysis between absolute values of GLS and LVEF and myocardial work parameters.

Parameters	GWI (mmHg%)		GCW (mmHg%)		GWE (mmHg%)		GWW (mmHg%)		SP (mmHg)		DP (mmHg)	
	*r*	*p*	*r*	*p*	*r*	*p*	*r*	*p*	*r*	*p*	*r*	*p*
GLS (%)	0.415	<0.05	0.374	<0.05	0.416	<0.05	−0.351	<0.05	−0.089	0.235	0.072	0.336
LVEF (%)	0.268	<0.05	0.401	<0.05	0.255	<0.05	−0.222	<0.05	−0.055	0.461	−0.054	0.476

GWI, global work index; GCW, global constructive work; GWE, global work efficiency; GWW, global wasted work; GLS, global longitudinal strain; LVEF, left ventricular ejection fraction; SP, systolic blood pressure; DP, diastolic blood pressure.

### Intra-observer and inter-observer reproducibility

The ICCs analyses for the intra- and inter-observer reproducibility of the myocardial work parameters derived from the PSL were shown in Table 6. All ICCs demonstrated a good to excellent reproducibility and the range of difference could be tolerated.

**Table 6:** Intra-observer and inter-observer reproducibility.

ICCs (95%CI)	GLS	GWI	GWE	GCW	GWW
Intra-observer	0.86 (0.79–0.90)	0.93 (0.89–0.95)	0.96 (0.94–0.97)	0.91 (0.86–0.94)	0.94 (0.91–0.96)
Inter-observer	0.89 (0.84–0.93)	0.91 (0.87–0.94)	0.92 (0.88–0.95)	0.87 (0.81–0.91)	0.90 (0.85–0.93)

ICC, intra-class correlation; GWI, global work index; GCW, global constructive work; GWE, global work efficiency; GWW, global wasted work; GLS, global longitudinal strain.

## Discussion

This was a comprehensive evaluation of left ventricular function in patients receiving KT by PSLs. The present study revealed several significant findings: (a) the left ventricular morphology and systolic function measured by conventional two-dimensional echocardiography showed significant improvement after KT; (b) the left ventricular myocardial work index measured by PSLs appeared significant improvement after KT, with some still lower than the control group; and (c) there was a significant correlation between left ventricular myocardial work indicators and GLS.

Patients with end-stage renal disease were under long-term exposure to uremic toxins, volume and pressure overload, hypertension, dyslipidemia, diabetes mellitus, arteriovenous fistula and so on. The above risk factors can lead to changes such as cardiac enlargement, left ventricular wall hypertrophy, myocardial edema, fibrosis and myocardial remodeling, resulting in a decrease in left ventricular systolic function and tension (22, 23). KT could reverse the above changes, manifested as an improvement in some measurement parameters of conventional echocardiography after surgery (24). And the reasons for improvement of LV function after KT were multifactorial and complementary, including removal of uremic toxins, reversal of LV, restoration of inflammation and hemodynamic changes (25). Eliminating the water overload and improving the composition of the inner environment lead to at least partial improvement of cardiac function, which was reflected mainly in favorable changes in LV indices (26).

LVEF is the most commonly used echocardiographic indicator for clinical evaluation of left ventricular systolic function and myocardial damage, however, its sensitivity is insufficient (27). Sometimes, although LVEF is within the normal range, there have been potential structure and function changes in the patient's hearts (28). 3D-STE is recommended for observing early myocardial damage, but it has a load dependence, which underestimates the true contractile capacity of the myocardium when the afterload increases (29, 30). However, one of the cardiovascular complications in KT patients is hypertension (31). Therefore, relying solely on LVEF or 3D-STE is difficult to accurately evaluate the true myocardial contractile function of KT patients with preserved ejection fraction (32).

Based on conventional echocardiography and 3D-STE, noninvasively PSLs is a new parameter, which has been investigated by studies in several cardiac conditions (33). PSLs replaced left ventricular pressure with brachial artery systolic pressure. What's more, it obtained the power integral of the left ventricular ejection period by multiplying the instantaneous left ventricular pressure and myocardial strain to represent non-invasive myocardial work (34). LV strain of longitudinal shortening as assessed by PSLs was predominantly influenced by subendocardial fibers. It was more suited to detect subtle changes of LV function than LVEF, which mainly depended on radial and circumferential deformation caused by mid-myocardial and epicardial fibers. By incorporating the left ventricular afterload into the left ventricular longitudinal strain analysis, the afterload dependent limitation of GLS and LVEF could be reduced, thus improving the accuracy of myocardial function assessment (35, 36).

Compared with the control group, patients with end-stage renal disease showed a significant decrease in GCW and GWE, a significant increase in GWW, and no significant change in GWI (37). This indicated that the systolic function of the left ventricle had undergone subclinical changes, which might be due to long-term exposure to uremia toxin, and some myocardial cells were fibrotic, resulting in mechanical electrical conduction obstruction (38). The continuous aggravation of left ventricular configuration, increasing renal toxicity, and weakening of myocardial contraction synchronization were not conducive to ejection, leading to a decrease in GCW. Although the ventricular myocardium increased myocardial work in combating increased afterload, unstable hemodynamic states and increased cardiac afterload could increase the loss of compensatory exercise energy in the myocardium, thereby increasing GWW (39, 40). The increase in myocardial energy loss lead to an increase in useless work and a decrease in effective work, resulting in a decrease in work efficiency and GWE (41, 42). The decline in GWI was speculated to be due to the weakened positive inotropic effect of nephrotoxin and increased afterload on the heart, as well as a decrease in tissue oxygen consumption under low metabolic conditions, which resulted in the heart doing more work to overcome afterload (43).

Some studies suggested that following KT, patients' creatinine and systolic blood pressure were still higher than control group, and the glomerular filtration rate, anemia, and nutritional status were worse than control group (44, 45). This might be due to the incomplete elimination of the long-term effects of uremic toxins after KT, the energy metabolism of myocardial cells had not yet recovered, and there was still edema and degeneration (46). PSLs showed that GWI and GCW had recovered, GWW and GWE increased after KT (47). The increase in GWI suggested that KT had increased the positive inotropic effect of the heart, while improving tissue metabolism and increasing oxygen consumption (48, 49). KT inhibited myocardial damage caused by uremic toxins and hypertension, leading to an increase in the effective work of myocardial contraction, that was, the GCW increased (50). The deformation, coordination, and synchronicity of the myocardium had restored, and the discrete dysfunction had reduced, resulting in a decrease in GWW. The increase in GCW and the decrease in GWW lead to an increase in GWE (51). GCW and GWI could reach normal levels after KT. Although GWW and GWE had improved after KT, there were still differences compared to the control group. This indicated that although KT could improve cardiac function and increase LVEF, there was still myocardial deformation damage, and left ventricular systolic function and myocardial remodeling had not been completely eliminated (52, 53). The above changes could not be detected using LVEF or GLS, while PSLs could be sensitively identified (45, 54).

Correlation analysis had found that GWI, GCW and GWE were positively correlated with the absolute values of GLS and LVEF, while GWW was negatively correlated with the absolute values of GLS and LVEF. This indicated that as myocardial damage worsened and myocardial contractility decreased, PSL could detect early myocardial contractile dysfunction in patients underwent dialysis, and the myocardial efficacy rate was not affected by blood pressure (55). The above findings were consistent with previous research results (56, 57).

However, antihypertensive medication did not significantly reduce after KT, suggesting that better hypertension control is not an independent factor in improving cardiac function after KT. Many issues on the pharmacological treatment of patients after KT are unclear or even controversial (58). For example, there are no studies comparing different classes of antihypertensive drugs, immunosuppressants or glucocorticoids after KT, therefore it is not known whether one class of medicine is better than another in patients underwent KT (59–61). Therefore, this is still a controversial issue and prospective trials are needed to resolve this debate.

## Limitation

Firstly, the relatively small cohort of participants and 12-month follow-up time is one of the limitations. Secondly, there might be differences between brachial artery blood pressure and the true pressure of the left ventricular cavity. We only investigated KT patients who received standard immunosuppressive therapy, without analyzing the differences in the effects of different immunosuppressive regimens on patients. The further research could expand the sample size and extend the follow-up time, as well as the effects of different immunosuppressive pathways, in order to improve the credibility and generalizability of the results and extend it to other transplant centers.

## Conclusion

In conclusion, noninvasive left ventricular PSLs include the afterload and strain indicators into the calculation at the same time, which can more accurately and sensitively evaluate the changes of KT on the myocardial performance of patients with end-stage renal disease. It provides more objective indicators for quantifying myocardial systolic function and studying myocardial mechanics, which is helpful for clinical diagnosis and follow-up reference.

## Data Availability

The raw data supporting the conclusions of this article will be made available by the authors, without undue reservation.
